# Pathogenesis, Virulence, and Infective Dose

**DOI:** 10.1371/journal.ppat.0030147

**Published:** 2007-10-26

**Authors:** Paul Schmid-Hempel, Steven A Frank

**Affiliations:** The Scripps Research Institute, United States of America

Some pathogens can begin an infection with only a small number of cells in the initial inoculum. For example, enterohemorrhagic strains of Escherichia coli require an infective dose of only about ten cells. By contrast, other pathogens, such as *Vibrio cholerae,* require a large number of cells (10^3^ to 10^8^ cells) in the inoculum to successfully infect a host (Figure 1).

**Figure 1:**
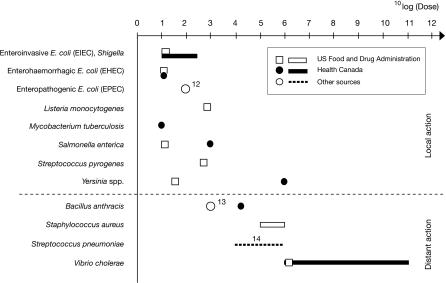
Dose and Mode of Pathogenic Mechanism Upper panel: bacteria with pathogenic mechanisms that depend on direct contact (local action). Lower panel: bacteria with pathogenic mechanisms that depend on secreted molecules (distant action). Dose information is from the US Food and Drug Administration [2] and Health Canada [3]. Various sources include references [12–14]. Values for *Yersinia* are considered low by the US Food and Drug Administration, while Health Canada refers to LD50 in mice.

We propose a new hypothesis to explain the wide diversity of infective dose among pathogens. In our hypothesis, the biochemical mechanisms of pathogenesis, which typically facilitate invasion by evading host immune defenses, explain much of the variation in infective dose. If these pathogenic mechanisms act locally, then we predict that the infective dose will be low; if the pathogenic molecules tend to diffuse and act at a distance, then we predict that the infective dose will be high. Local action requires relatively few molecules and therefore relatively few infecting cells. Distant action requires that the infecting population of pathogens builds up sufficient quantities of diffusible molecules to achieve sufficient effect before the host can clear the infection (the “frontal attack strategy” described in [1]); thus, larger infecting doses are required to produce sufficient concentrations of the diffusible pathogenic molecules. Few standardized studies of infective dose have been conducted, in part because there has never been a clear conceptual framework within which to study the problem and formulate testable predictions. To test our hypothesis, we therefore use the reasonably standardized databases of the United States Food and Drug Administration [2] and Health Canada [3], supplemented by additional references.

The diverse and well-defined secretion systems of bacteria provide good contrasts between local and distant mechanisms of pathogenesis. For local action, the upper part of Figure 1 shows several examples in which the bacteria either directly transfer proteins into the host cells (via a type III secretion system) or act via bacterial surface-bound molecules. Local molecular mechanisms are illustrated by the following examples [4,5]: *Shigella* infection depends on direct injection of several bacterial proteins (invasion plasmid antigens) via a type III secretion system [5], by which the host cell surface is manipulated and the bacterium is taken up into a vacuole. Enterohemorrhagic E. coli infects using a “attaching and effacing” mechanism, which is mediated by an outer-membrane protein (intimin) or by direct delivery of receptors and proteins inducing host cell cytoskeleton changes [6]. Similarly, enteropathogenic *E. coli* relies on direct delivery of receptors and additional proteins [4,6] by a type III secretion system. The bacteria-produced receptor is delivered allowing for the binding with further bacterial protein that manipulates the host cell. Listeria monocytogenes invades cells of intestinal mucosa or macrophages with the help of bacterial membrane-bound proteins (internalins) [4,5,7]. Forced phagocytosis puts the bacterium inside host cell vacuoles. The other bacteria in the upper part of Figure 1 display similar local action of pathogenesis.

The lower part of Figure 1 shows four cases of distant action and, as we predict, relatively high numbers of pathogens required in the inoculum to initiate an infection. We classify the species as having distant action if a necessary component of invasion and pathogenesis includes a secreted factor that diffuses before exerting its pathogenic effect. This may arise, for example, through immune modulators delivered by the general secretory pathway, or the type I and type II secretory systems in Gram-negative bacteria. For *V. cholerae,* the action of the secreted cholera toxin is necessary and likely to be the limiting step, even though the parasite also uses a locally acting type IV secretion system that delivers the bacteria-produced receptor for cholera toxin directly into the host cell [4,5]. Infection by Bacillus anthracis depends on the bacterial anthrax toxin, of which one essential component (protective agent) is secreted by the bacteria. After the protective agent diffuses and binds to receptors of host immune cells, the other two components (lethal factor and edema factor) are transported directly into host cells [8,9] and help evade host immunity. Staphylococcus aureus has an astonishing variety of both locally acting (surface-bound) and secreted, diffusible factors; however, successful infection requires sufficient quantity of the initially secreted, immune-modulating proteins [10]. Streptococcus pneumoniae relies on a cytoplasmic protein secreted by a cell-bound autolysin, inducing pore formation in host mucosal cells [5,11].

In this article, we emphasized the distinction between local and distant action of pathogenic mechanisms with regard to invasion and other criteria relevant for the observed dose-response variation among pathogens. This distinction between local and distant action may also explain some of the differences between pathogens in the total amount of harm (virulence) caused by infection. For example, all of the pathogens listed in Figure 1 can cause severe disease, but it is remarkable that those pathogens that secrete distantly acting immune modulators include most of the extremely virulent pathogens, such as B. anthracis or *S. aureus.* Hence, this framework should also lead to a better understanding of the evolution of virulence itself. Previously, no broadly applicable and predictive hypothesis of infective dose existed, so few studies have been designed explicitly to study the problem. We present the examples in Figure 1 to illustrate our hypothesis and to show that a preliminary survey supports the predictions. With our clear predictions, new studies on bacteria and on viruses can directly test the idea that local versus distant action influences infective dose and virulence. 

## Supporting Information

### Accession Numbers

Information on genetic sequences for the mentioned proteins can be found in GenBank (http://www.ncbi.nlm.nih.gov/entrez/): *Shigella flexeneri* (IpaB, 1238055; IpaC, 1238054); Escherichia coli intimin (915471); Listeria monocytogenes internalins (InlA, 985151; InlB, 98692); Vibrio cholerae (O139, ctxA, ctxB: X76391); Bacillus anthracis (protective agent, CDS AF306780; lethal factor, CDS AY997300; edema factor, CDS AY997301); Streptococcus pneumoniae autolysin (933669).

## References

[ppat-0030147-b001] Merrell DS, Falkow S (2004). Frontal and stealth attack strategies in microbial pathogenesis. Nature.

[ppat-0030147-b002] United States Food and Drug Administration (2003). The bad bug book.

[ppat-0030147-b003] Health Canada (2003). Material safety data sheets.

[ppat-0030147-b004] Donnenberg MS (2000). Pathogenic strategies of enteric bacteria. Nature.

[ppat-0030147-b005] Salyers AA, Whitt DD (2002). Bacterial pathogenesis. 2nd edition.

[ppat-0030147-b006] Kaper JB, Nataro JP, Mobley HL (2004). Pathogenic Escherichia coli. Nat Rev Microbiol.

[ppat-0030147-b007] Dussurget O, Pizarro-Cerda J, Cossart P (2004). Molecular determinants of Listeria monoytogenes virulence. Annu Rev Microbiol.

[ppat-0030147-b008] Moayeri M, Leppla S (2004). The roles of anthrax toxin in pathogenesis. Curr Opin Microbiol.

[ppat-0030147-b009] Abrami L, Reig N, Gisou van der Goot F (2005). Anthrax toxin: The long and winding road that leads to the kill. Trends Microbiol.

[ppat-0030147-b010] Rooijakkers SHM, van Kessel KPM, van Strijp JAG (2005). Staphylococcal innate immune evasion. Trends Microbiol.

[ppat-0030147-b011] Mitchell TJ (2003). The pathogenesis of streptococcal infections: from tooth decay to meningitis. Nat Rev Microbiol.

[ppat-0030147-b012] Teunis PFM, Takumi K, Shinagawa K (2004). Dose response for infection by Escherichia coli O157:H7 from outbreak data. Risk Analysis.

[ppat-0030147-b013] Sewell DL (1995). Laboratory-associated infections and biosafety. Clin Microbiol Rev.

[ppat-0030147-b014] Yershov AL, Jordan BS, Guymon CH, Dubick MA (2005). Relationship between the inoculum dose of Streptococcus pneumoniae and pneumonia onset in a rabbit model. Eur Respir Res.

